# Atorvastatin-induced senescence of hepatocellular carcinoma is mediated by downregulation of hTERT through the suppression of the IL-6/STAT3 pathway

**DOI:** 10.1038/s41420-020-0252-9

**Published:** 2020-03-30

**Authors:** Sin-Ting Wang, Shi-Wei Huang, Kuang-Ting Liu, Teng-Yu Lee, Jeng-Jer Shieh, Chun-Ying Wu

**Affiliations:** 1 Division of Translational Research and Center of Excellence for Cancer Research, Taipei Veterans General Hospital, Taipei, Taiwan; 2 Center for Cell Therapy and Translation Research, China Medical University Hospital, Taichung, Taiwan; 3 Department of Biomedical Sciences, National Chung Hsing University, Taichung, Taiwan; 4 Department of Pathology & Laboratory Medicine, Taoyuan Armed Forces General Hospital, Taoyuan, Taiwan; 5 Division of Gastroenterology and Hepatology, Taichung Veterans General Hospital, Taichung, Taiwan; 6 Department of Medicine, Chung Shan Medical University, Taichung, Taiwan; 7 Department of Education and Research, Taichung Veterans General Hospital, Taichung, Taiwan; 8 Department of Life Sciences and Rong Hsing Research Center for Translational Medicine, National Chung Hsing University, Taichung, Taiwan; 9 Institute of Biomedical Informatics, Institute of Clinical Medicine, and Institute of Public Health, National Yang-Ming University, Taipei, Taiwan; 10 Department of Public Health, China Medical University, Taichung, Taiwan; 11 National Institute of Cancer Research, National Health Research Institutes, Miaoli, Taiwan; 12 Taiwan Microbiota Consortium, Taipei, Taiwan

**Keywords:** Cancer therapy, Senescence

## Abstract

Hepatocellular carcinoma (HCC), a hepatic malignancy, has a poor prognosis and contributes to cancer-related death worldwide. Cellular senescence is an anticancer therapeutic strategy that causes irreversible cell cycle arrest and enables immune-mediated clearance of cancer cells. Atorvastatin, an HMG-CoA reductase inhibitor, has been shown to inhibit tumor growth and induce apoptosis or autophagy in malignant tumors. However, whether atorvastatin can induce HCC cell senescence and the mechanisms involved are poorly understood. The effects of atorvastatin-induced senescence were examined in both HCC cells and mouse xenograft models. The phenomenon and mechanism of senescence were examined by cell cycle analysis, senescence-associated β-galactosidase (SA-β-gal) staining and western blotting in HCC cells, and HCC tissues from mice were analyzed by immunohistochemical (IHC) staining. We demonstrated that atorvastatin induced cell growth inhibition and G0/G1 phase cell cycle arrest, leading to senescence in HCC cells. Atorvastatin-induced senescence was independent of p53, p14, and p16, and atorvastatin not only decreased the secretion of IL-6, a major senescence-associated secretory phenotype (SASP) factor, and the phosphorylation of STAT3 but also inhibited the expression of hTERT, a catalytic subunit of telomerase. Supplementation with exogenous IL-6 reversed both atorvastatin-induced suppression of STAT3 phosphorylation and hTERT expression and atorvastatin-induced senescence. Overexpression of constitutively activated STAT3 rescued HCC cells from atorvastatin-induced hTERT suppression and senescence. Moreover, atorvastatin decreased tumor growth in mouse xenograft models. Consistent with these results, atorvastatin decreased the IL-6, p-STAT3, and hTERT levels and increased β-gal expression in tumor sections. Taken together, these data indicate that atorvastatin can induce atypical cellular senescence in HCC cells to inhibit tumor growth, an effect mediated by downregulation of hTERT through suppression of the IL-6/STAT3 pathway.

## Introduction

Hepatocellular carcinoma (HCC) is the most common malignant liver cancer with a poor prognosis and is responsible for many cancer-related deaths worldwide^1,2^. HCC usually initiates and develops under inflammatory conditions, such as hepatitis B virus infection, hepatitis C virus infection, alcoholic liver disease, nonalcoholic steatohepatitis, and metabolic syndromes, in conjunction with cirrhosis^3^. As a result, the most important risk factor for HCC progression is long-term chronic liver inflammation, which causes liver damage; this damage greatly contributes to HCC development.

Cellular senescence is an important factor that restricts tumor growth and modulates cancer-associated immune responses. Cellular senescence is a state of irreversible cell cycle arrest in response to stress conditions, such as DNA damage, telomere dysfunction, chromatin disruption, and oncogene activation^4,5^. The phenotypic hallmarks of cellular senescence include an enlarged, flattened morphology; increased granularity, and the expression of senescence-associated β-galactosidase (SA-β-gal)^6–8^. In addition, senescent cells display the senescence-associated secretory phenotype (SASP), which corresponds to the secretion of various cytokines, chemokines, growth factors, and proteases that affect the surrounding cells; furthermore, the SASP has been reported to have antitumorigenic effects^9,10^. Thus the induction of senescence in cancer cells has been suggested as a therapeutic strategy.

Statins, which are 3-hydroxy-3-methylglutaryl coenzyme A reductase inhibitors that effectively decrease serum cholesterol levels and reduce the incidence of cardiovascular events, are widely used to treat patients with hypercholesterolemia^11^. In addition, several previous studies have shown that the potential mechanisms of statins include inhibition of tumor cell proliferation, promotion of cell cycle arrest, induction of apoptosis, and inhibition of cancer cell metastasis^12,13^. Our previous study indicated that simvastatin was associated with improved overall survival in HCC patients. Simvastatin exerted its chemotherapeutic effect in HCC by inducing G0/G1 cell cycle arrest via a novel mechanism through which statins upregulate p21 and p27 by activating the AMPK pathway and inhibiting the signal transducer and activator of transcription factor 3 (STAT3)-Skp2 pathway, respectively^13^. Atorvastatin has been found to inhibit tumor growth and to induce apoptosis or autophagy in malignant tumors^14–18^. However, a few studies stated that atorvastatin could potentially modulate cellular senescence and reduce tumor formation in HCC^19^.

In the present study, we discovered that atorvastatin induced cell growth inhibition and cell cycle arrest to cause senescence in HCC cells. We demonstrated that the involvement of interleukin (IL)-6 in atorvastatin-induced cellular senescence was mediated by the regulation of hTERT through the IL-6/STAT3 signaling pathway. Furthermore, we established a HepG2 tumor-bearing xenograft mouse model to examine the antitumor effect of atorvastatin in vivo and observed that atorvastatin suppressed tumor growth and that the expression patterns of IL-6, STAT3, TERT, and β-gal, along with β-gal activity, were similar to those observed in in vitro studies. Taken together, these results indicate that the induction of senescence-like cell growth arrest by atorvastatin is related to inhibition of the IL-6/STAT3/TERT pathway. These findings indicate the potential differential impact of atorvastatin and a novel molecular mechanism of promoting senescence for adjuvant treatment of HCC.

## Results

### Atorvastatin caused G0/G1 arrest in HCC cells

**Fig. 1:**
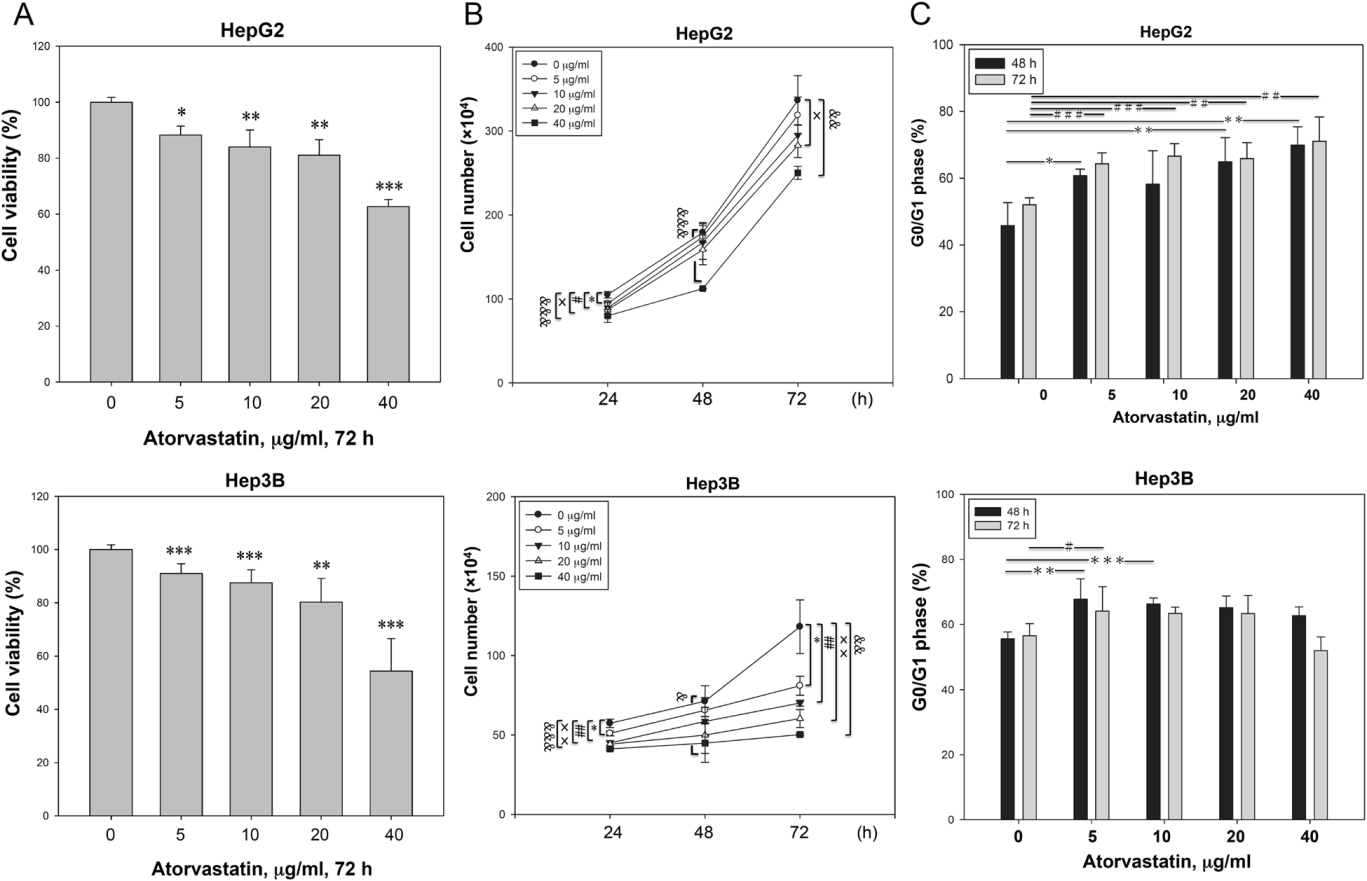
Atorvastatin induced growth inhibition and G0/G1 phase arrest in HCC cells. Atorvastatin suppressed the growth (**a**) and decreased the total numbers (**b**) of HepG2 and Hep3B cells. The data are presented as the percentages of vehicle-treated cells. **c** Atorvastatin induced G0/G1 arrest in HepG2 and Hep3B cells. Cells were treated with atorvastatin (0, 5, 10, 20, or 40 μg/ml) for 24, 48, or 72 h, and cell growth was then measured by the CCK-8 assay (**a**). Cell numbers were determined by counting viable cells (**b**), and cell cycle distributions were measured via flow cytometry (**c**). **b** The asterisk indicates a significant difference between control and 5 μg/ml; the pound sign indicate a significant difference between control and 10 μg/ml; the double dagger indicates a significant difference between control and 20 μg/ml; and the ampersand indicates a significant difference between control and 40 μg/ml. **c** The asterisk indicates a significant difference between untreated and treated for 48 h; the pound sign indicates a significant difference between untreated and treated for 72 h.

To examine the effect of atorvastatin on the growth of HCC cells, we measured the viability and growth rate of p53-wild-type HepG2 and p53-null Hep3B cell lines. As shown in Fig. 1a, atorvastatin inhibited cell proliferation in a dose-dependent manner, and the growth rate of atorvastatin-treated HCC cells was relatively slower than that of untreated HCC cells in a dose-dependent manner at various time points (Fig. 1b). Next, we investigated whether cell growth inhibition is associated with cell cycle interference and found that the population of cells in G0/G1 phase was increased in all atorvastatin-treated cells compared to that in control cells (Fig. 1c). A similar but less pronounced effect on cell cycle arrest was also found in Hep3B cells treated with a low dose of atorvastatin (Fig. 1c). In addition, treatment with a high dose of atorvastatin (40 μg/ml) not only caused cell cycle arrest but also induced apoptosis in both HCC cell lines, especially in Hep3B cells, after incubation for 72 h (Supplementary Fig. 1a, b). The results indicated that the low dose of atorvastatin reduced the growth rate and caused G0/G1 arrest in HCC cells.

### Atorvastatin induced senescence in HCC cells

**Fig. 2:**
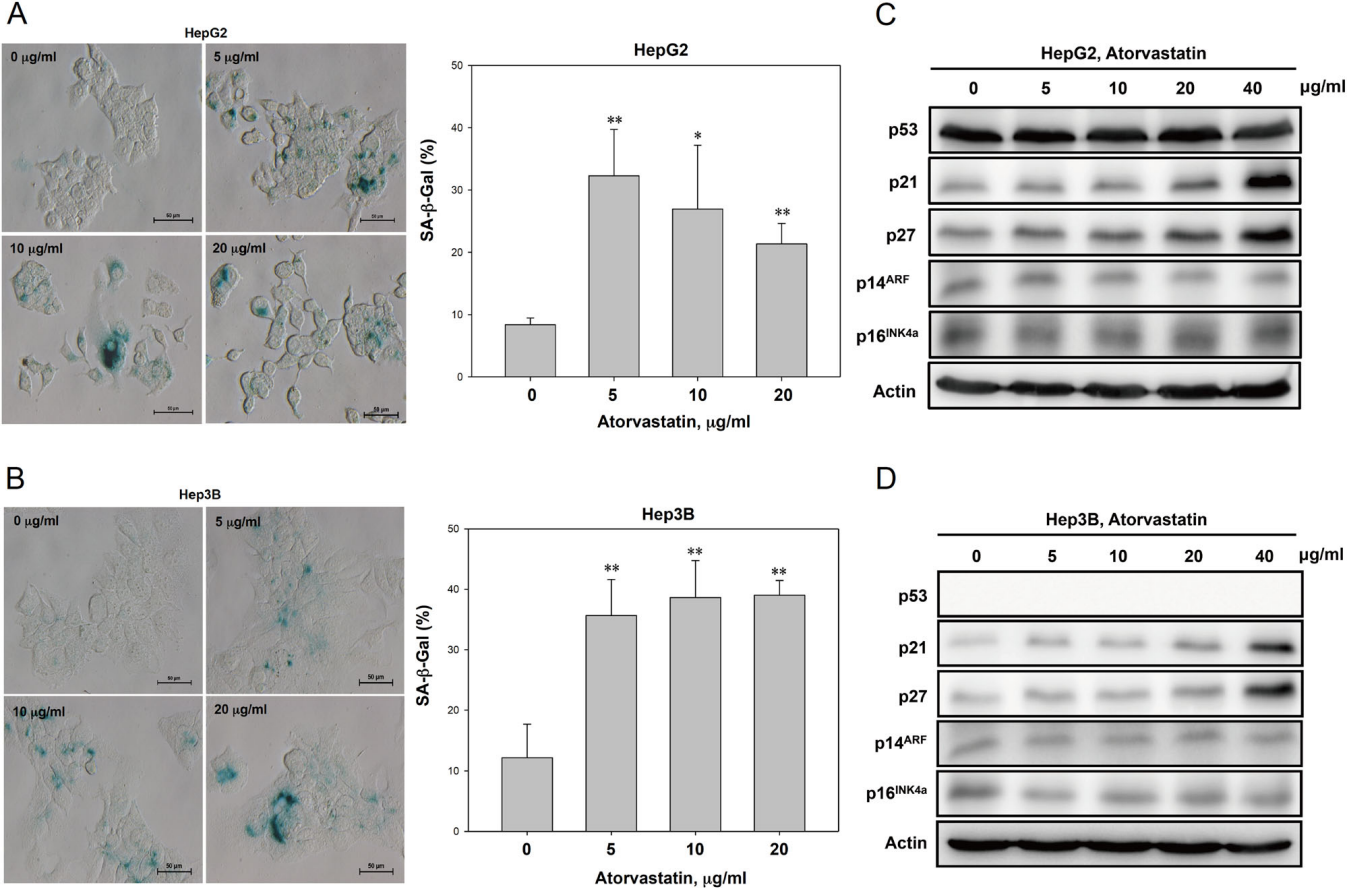
Atorvastatin induced senescence in HCC cells. **a**, **b** Atorvastatin induced senescence-associated β-galactosidase (SA-β-gal) activity in HepG2 and Hep3B cells. **c**, **d** Atorvastatin treatment of p53-wild-type HepG2 and p53-null Hep3B cells induced p21 and p27 expression but did not alter the expression of p14^ARF^ or p16^INK4a^. HepG2 and Hep3B cells were treated with atorvastatin (0, 5, 10, or 20 μg/ml) for 72 h and then stained for SA-β-gal (**a**, **b**) and subjected to immunoblotting (**c**, **d**).

We hypothesized that atorvastatin treatment could trigger senescence in HCC cells. We calculated the percentage of senescent HepG2 and Hep3B cells via SA-β-gal staining to determine whether atorvastatin induces senescence in HCC cells. The number of senescent HepG2 and Hep3B cells treated with low dose of atorvastatin for 72 h was significantly higher than that of the corresponding untreated cells (Fig. 2a, b). Previous studies indicated that increased expression of p14^ARF^ and p16^INK4a^ promote cellular senescence and maintain suppression of cell growth^8,20–24^. However, despite the induction of G0/G1 cell cycle arrest and upregulation of p21 and p27 expression after atorvastatin treatment, atorvastatin did not significantly derepress the expression of p14^ARF^ and p16^INK4a^ in either the p53-wild-type HepG2 or p53-null Hep3B cell lines (Fig. 2c, d). Taken together, our data suggest that atorvastatin induced senescence in HCC cells and that p53, p14^ARF^, and p16^INK4a^ may not be involved in this process.

### IL-6 counteracted atorvastatin-induced senescence in HCC cells

**Fig. 3:**
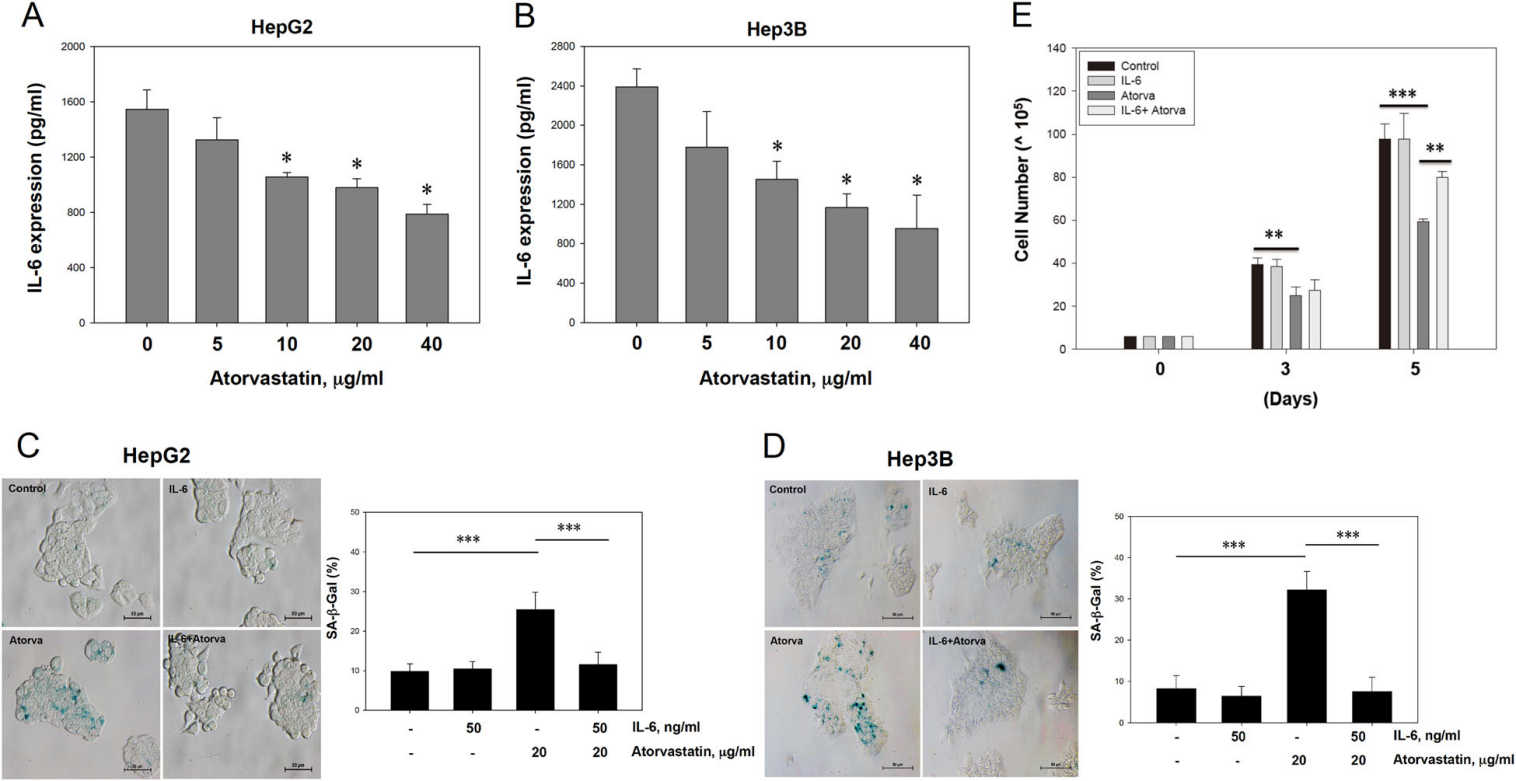
IL-6 secretion was decreased by atorvastatin treatment and was involved in atorvastatin-induced cellular senescence in HCC cells. **a**, **b** Atorvastatin suppressed IL-6 secretion in HepG2 and Hep3B cells. Cells were treated with various concentrations of atorvastatin for 72 h. Culture supernatants were collected during treatment, and IL-6 concentrations were measured by ELISA. **c**, **d** Pretreatment with IL-6 and the addition of exogenous IL-6 every 24 h rescued HCC cells from atorvastatin-induced senescence. **e** Cell growth, which was arrested after atorvastatin treatment, was restored in HCC cells pretreated with IL-6 and administered exogenous IL-6. IL-6 (50 ng/ml) was administered to HCC cells before and during atorvastatin treatment (**c**, **d**) or for 3 or 5 days (**e**), and cells were then subjected to SA-β-gal staining (**c**, **d**) and counted to determine the viable population (**e**).

Cellular senescence is often accompanied by acquisition of the SASP. High levels of IL-6, a major SASP factor, promote the progression of HCC and other types of liver cancer^25–27^. In addition, statins have been reported to reduce IL-6 production in smooth muscle cell (SMC)/monocyte cocultures^28^. Thus evaluation of IL-6 levels in HCC cells exhibiting atorvastatin-induced senescence is interesting. Compared to untreated cells, HepG2 and Hep3B cells treated with atorvastatin showed reduced levels of IL-6 in the supernatant (Fig. 3a, b). Next, both IL-6 pretreatment and exogenous addition of IL-6 to atorvastatin-treated HCC cells every 24 h revealed that, compared with atorvastatin treatment alone, combined IL-6 and atorvastatin treatment significantly decreased the number of SA-β-gal-positive HepG2 and Hep3B cells (Fig. 3c, d). In addition, cell growth arrest was recovered (Fig. 3e). These data indicated that atorvastatin induces senescence in HCC cells, with a distinct SASP and reduced IL-6 levels, and that IL-6 might play a counteracting role in atorvastatin-induced senescence in HCC cells.

### Atorvastatin downregulated hTERT expression and STAT3 activation to affect senescence in HCC cells

**Fig. 4:**
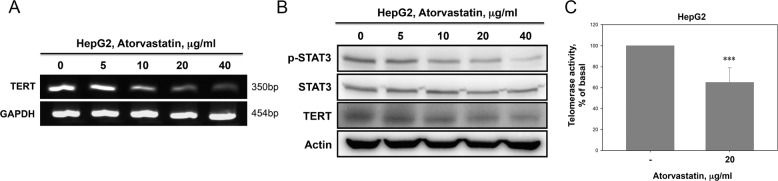
Atorvastatin inhibited hTERT expression, telomerase activity, and STAT3 activation in HepG2 cells. **a** Cells treated with atorvastatin exhibited significantly decreased TERT mRNA expression. **b** Cells treated with atorvastatin exhibited decreased TERT protein expression and STAT3 phosphorylation. HepG2 cells were treated with atorvastatin (0, 5, 10, 20, and 40 μg/ml) for 72 h, and mRNAs and proteins were then isolated and analyzed by RT-PCR (**a**) and immunoblotting (**b**), respectively. **c** Atorvastatin inhibited telomerase activity in HepG2 cells. Telomerase activity was assessed in HepG2 cells treated with 20 μg/ml atorvastatin for 72 h. The data are expressed as the percentage of telomerase activity.

Telomerase plays a critical role in of cancer progression via maintenance of genome integrity and regulation of senescence. The reverse transcriptase catalytic subunit (TERT) is a major part of the telomerase enzyme and is expressed only in immortalized cells and stem cells^29^. STAT3 has been reported to directly regulate TERT expression in several types of human cancer cells by binding to the promoter region of TERT^30^. Thus we investigated whether atorvastatin contributed to the downregulation of TERT and STAT3 expression in HCC cells. As shown in Fig. 4a, the TERT mRNA level was significantly decreased in atorvastatin-treated HepG2 cells compared to untreated cells. Atorvastatin also decreased the TERT and p-STAT3 protein levels under the same conditions (Fig. 4b). Furthermore, compared with nontreated cells, atorvastatin-treated cells exhibited telomerase activity inhibition, as shown in Fig. 4c. These findings suggested that atorvastatin inhibited TERT expression and STAT3 activation in HepG2 cells.

### Atorvastatin-induced senescence was mediated by downregulation of hTERT via suppression of the IL-6/STAT3 pathway

**Fig. 5:**
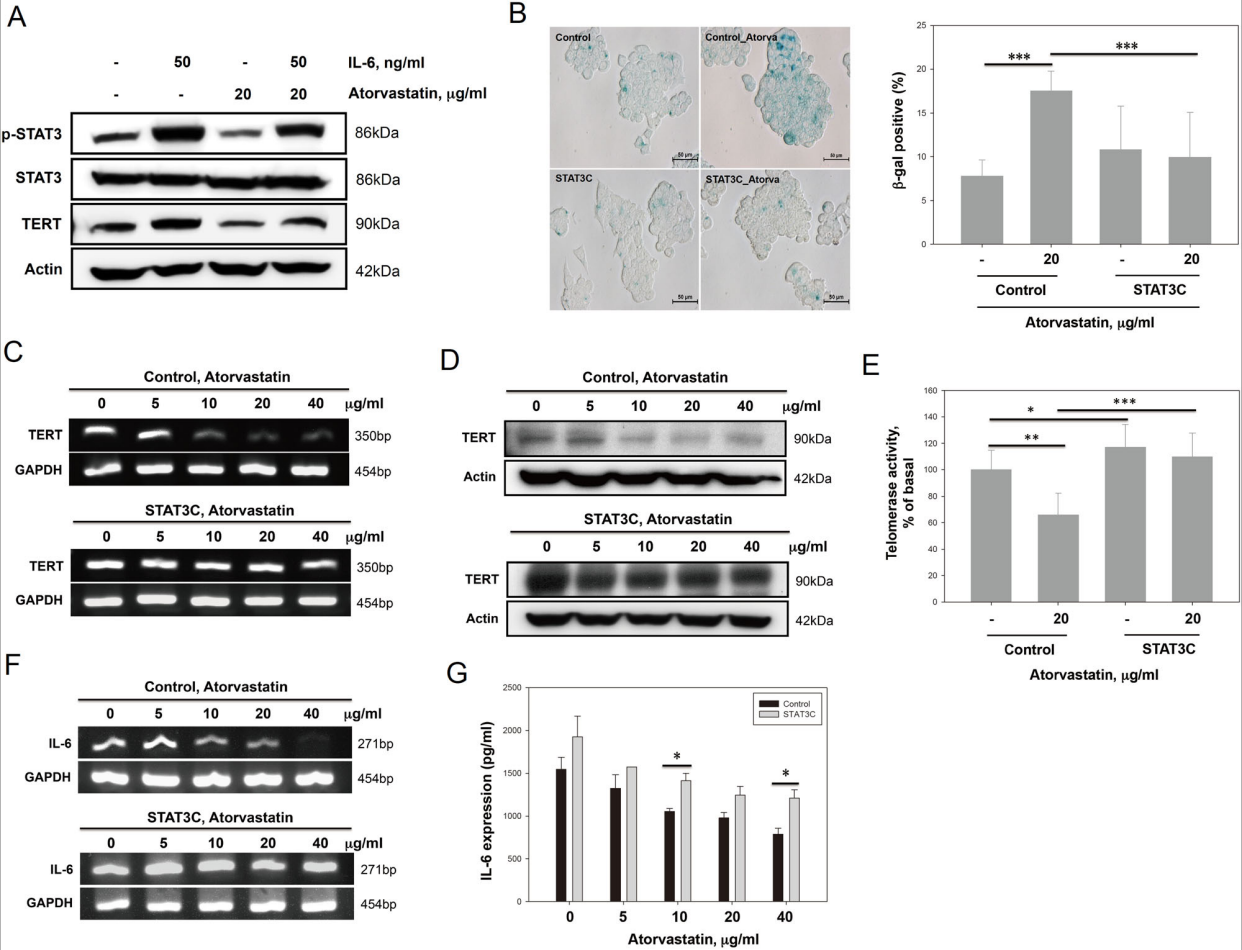
Atorvastatin downregulated TERT expression by inhibiting IL-6/STAT3 signaling to induce senescence in HepG2 cells. **a** The levels of TERT and p-STAT3 proteins were restored upon the addition of exogenous IL-6 to atorvastatin-treated HepG2 cells. Then 50 ng/ml IL-6 was added back to atorvastatin-treated cells for 72 h, and the cells were then subjected to immunoblotting. **b** Overexpression of constitutively active STAT3 protected HepG2 cells from atorvastatin-induced cellular senescence. HepG2 cells overexpressing a constitutively active mutant of STAT3 (STAT3C) and control HepG2 cells were cultured in the presence or absence of atorvastatin (20 μg/ml) for 72 h, and then senescence was evaluated by SA-β-gal staining. **c**, **d** The TERT mRNA (**c**) and protein expression levels (**d**) in STAT3C-overexpressing HepG2 cells were not decreased compared with those in control HepG2 cells after atorvastatin treatment. STAT3C-overexpressing and control HepG2 cells were treated with atorvastatin (0, 5, 10, 20, and 40 μg/ml) for 72 h, and mRNA and protein were then isolated and analyzed by RT-PCR (**c**) and immunoblotting (**d**), respectively. **e** Overexpression of STAT3C in HepG2 cells reversed atorvastatin-mediated downregulation of telomerase activity. Telomerase activity was measured in STAT3C-overexpressing and control HepG2 cells treated with 20 μg/ml atorvastatin for 72 h. **f**, **g** The levels of IL-6 mRNA (**f**) and secreted IL-6 (**g**) were restored in STAT3C-overexpressing HepG2 cells treated with atorvastatin. STAT3C-overexpressing and control HepG2 cells were treated with atorvastatin (0, 5, 10, 20, and 40 μg/ml) for 72 h. Then, after the cell culture supernatant was collected, mRNA was extracted, and RT-PCR and IL-6 ELISA assays were performed.

The data in Fig. 3c, d show that the effects of atorvastatin on HCC cell senescence were reduced by exogenous IL-6. Next, we evaluated whether TERT and STAT3 were involved in the mechanism by which IL-6 counteracts atorvastatin-induced senescence in HepG2 cells. First, we found that IL-6 increased the levels of phosphorylated STAT3 and TERT protein. Furthermore, the levels of TERT and phosphorylated STAT3 in atorvastatin-treated HepG2 cells were restored by extracellular IL-6 supplementation (Fig. 5a). Second, as shown in Fig. 5b, overexpression of constitutively activated STAT3 (STAT3C) reversed atorvastatin-induced senescence in HepG2 cells. Consistent with these results, STAT3C overexpression in atorvastatin-treated HepG2 cells rescued the effects of TERT mRNA and protein downregulation and the decrease in telomerase activity (Fig. 5c–e). Importantly, after atorvastatin treatment, the IL-6 mRNA expression levels in STAT3C-transfected cells were not significantly decreased (Fig. 5f), although the level of IL-6 in the supernatant of STAT3C-transfected cells was slightly decreased (Fig. 5g). Taken together, our findings suggest that atorvastatin may downregulate TERT expression by inhibiting the IL-6/STAT3 autocrine loop to induce senescence in HepG2 cells.

### Atorvastatin inhibited tumor growth in HCC xenograft animal models

**Fig. 6:**
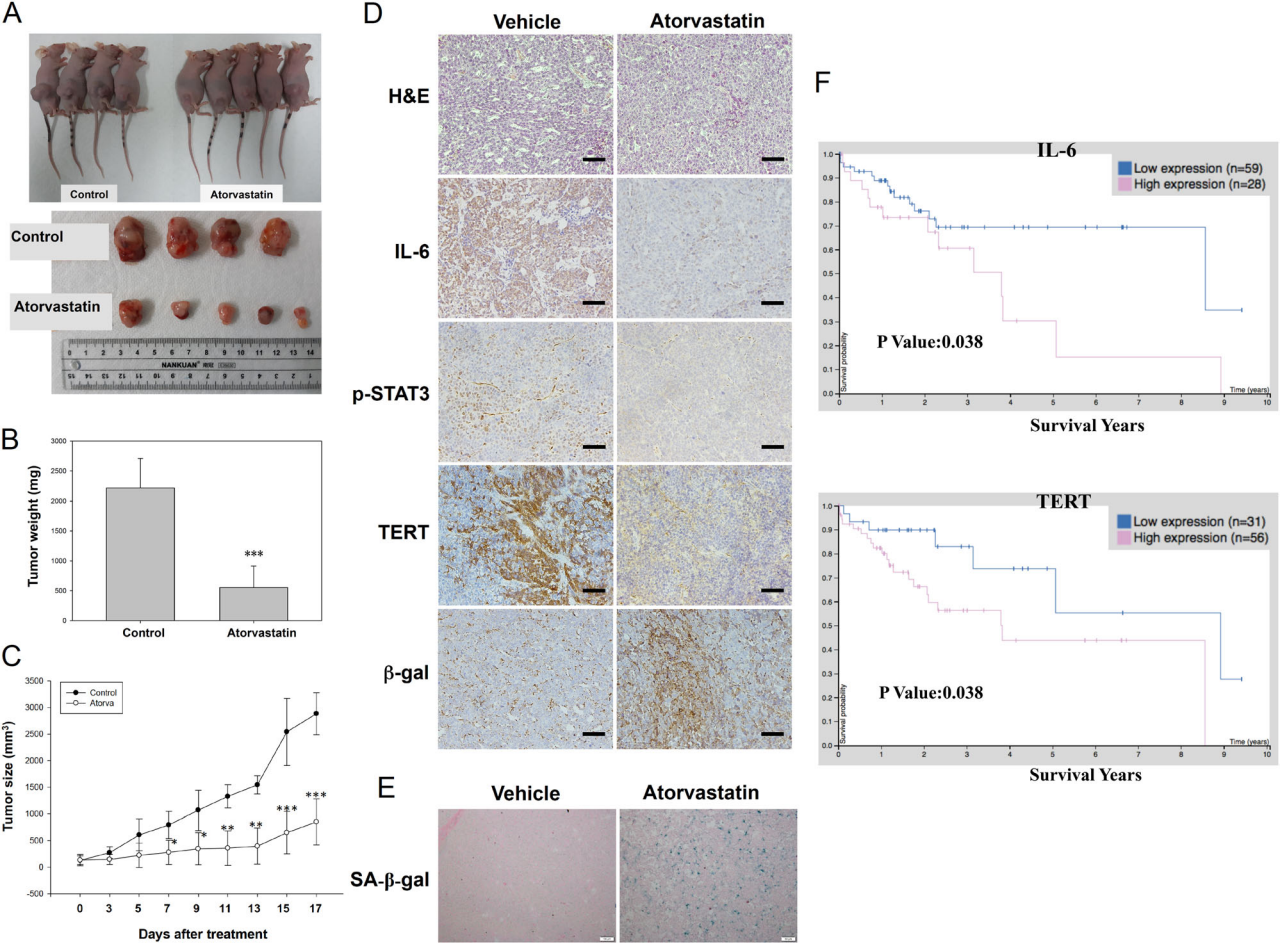
Atorvastatin suppressed HepG2 tumor growth in xenograft mice. A tumor-bearing mouse model was established by subcutaneously inoculating a suspension of HepG2 cells into the flanks of BALB/c nude mice. HepG2 tumor-bearing mice were treated with vehicle or atorvastatin (20 mg/kg) twice daily by intraperitoneal injection. **a** Mice in the vehicle and atorvastatin treatment groups were sacrificed 17 days after the last atorvastatin injection. Tumor tissues were harvested from each mouse. **b** Tumor weights were measured after sacrifice. **c** Growth curves of tumors excised from vehicle- and atorvastatin-treated mice. Tumor volumes were calculated twice daily for 17 days. The results are shown as the means ± S.E.M; vehicle-treated mice were compared to atorvastatin-treated mice. *p < 0.05, **p < 0.01, ***p < 0.001. **d**, **e** IHC analysis using antibodies against human IL-6, p-STAT3, TERT, and β-gal (**d**) and measurement of SA-β-gal activity (**e**) in tumor tissues from vehicle- and atorvastatin-treated mice. All scale bars represent 50 μm. **f** Increased IL-6 and TERT mRNA expression was significantly associated with poor overall survival in patients with HCC. Clinical data for patients with liver cancer were obtained from a TCGA dataset in The Human Protein Atlas (https://www.proteinatlas.org/).

To investigate HCC cell senescence after atorvastatin treatment in vivo, we established a HepG2 tumor-bearing animal model. As shown in the images in Fig. 6a, the tumor sizes were significantly decreased in the atorvastatin-treated group compared to the vehicle-treated group; in addition, the tumor weights were lower (Fig. 6b), and tumor growth was suppressed (Fig. 6c) in atorvastatin-treated HepG2 xenograft mice. Consistent with these results, comparison of tumor tissues between the vehicle- and atorvastatin-treated groups showed that the levels of IL-6, p-STAT3, and TERT were decreased in mice treated with atorvastatin (Fig. 6d). Moreover, atorvastatin treatment increased β-gal expression and SA-β-gal activity in tumor tissues (Fig. 6e). To examine whether high expression of IL-6 and TERT in patients with HCC was associated with poor prognosis, we analyzed a The Cancer Genome Atlas dataset from The Human Protein Atlas (https://www.proteinatlas.org/). As shown in Fig. 6f, increased IL-6 and hTERT expression was correlated with poor overall survival probability in 87 HCC patients. Taken together, these data suggest that high expression of IL-6 and hTERT may contribute to poor outcomes in patients with HCC and that pharmacological inhibition of the IL-6/STAT3/TERT signaling axis with atorvastatin may improve patient outcomes by inducing HCC cell senescence in vivo.

## Discussion

**Fig. 7:**
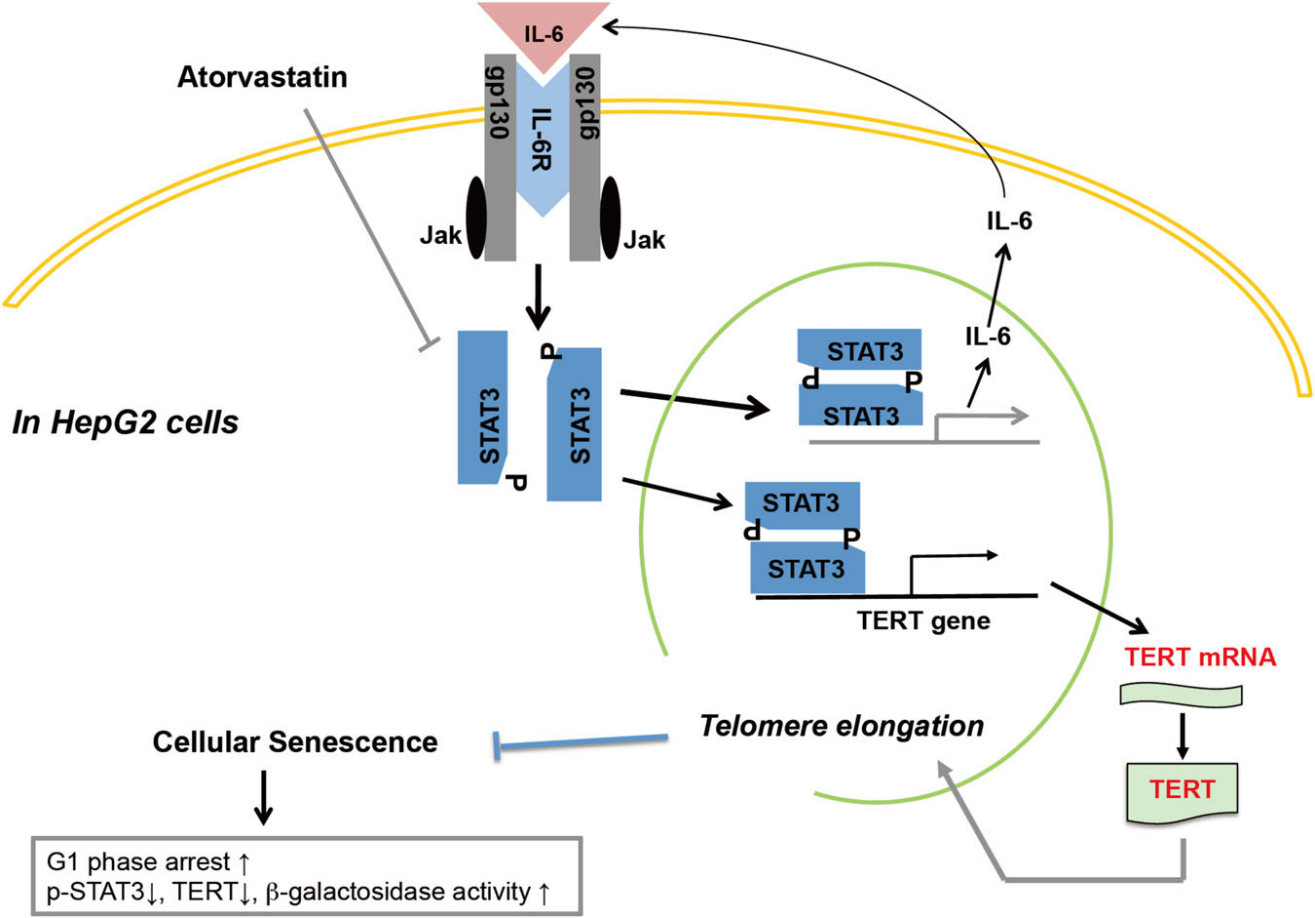
Summary of the in vitro and in vivo results of this study. Atorvastatin inhibited STAT3 phosphorylation to decrease IL-6 production and TERT expression. The decrease in IL-6 production attenuated the STAT3/IL-6 positive feedback loop and decreased TERT expression, inducing senescence in HCC cells.

In this study, we demonstrated that atorvastatin induced senescence in HCC cells independent of p53, p14, and p16. Atorvastatin not only decreased IL-6 secretion and STAT3 activation but also inhibited hTERT expression, which downregulates telomerase activity (Fig. 7). Supplementation with exogenous IL-6 and overexpression of STAT3C reversed atorvastatin-induced suppression of hTERT expression and senescence. Moreover, atorvastatin treatment decreased tumor growth and weight in an HCC xenograft animal model, which were associated with decreased IL-6 and TERT expression and STAT3 phosphorylation and increased β-gal expression and SA-β-gal activity in tumors. Thus atorvastatin-induced cellular senescence might occur by suppressing the IL-6/STAT3/TERT signaling pathway in HCC.

Cellular senescence is regarded as a signal transduction process leading to the irreversible arrest of cell proliferation and is accompanied by changes in the cellular phenotype. Recent studies have reported that statins have dual roles in the regulation of cellular senescence^31,32^. In endothelial progenitor cells, both atorvastatin and mevastatin reduced cellular senescence by modulating genes involved in the cell cycle, including upregulation of cyclins and downregulation of p27^Kip1^^31^. In cancer cells, lovastatin induced prostate cancer cell senescence by modulating RhoA expression^33^, and simvastatin increased reactive oxygen species production and promoted senescence via activation of the p53/p21 pathway in melanoma cells^34^. In cancer-associated fibroblasts, simvastatin inhibited protein prenylation to decrease the SASP of senescent human fibroblasts, which mitigated the effects of conditioned medium from senescent cells on breast cancer cell proliferation and endocrine resistance^32^. Therefore, statins have opposing effects: inducing senescence in cancer cells and suppressing senescence in normal or nonmalignant cells. Here we observed that atorvastatin caused cell growth inhibition and G0/G1 phase arrest (Fig. 1) and increased the population of senescent SA-β-gal-positive cells (Fig. 2a, b) independent of p14^ARF^ and p16^INK4A^ expression in p53-wild-type HepG2 and p53-null Hep3B cells (Fig. 2c, d). These data indicate that atorvastatin-induced senescence in HCC cells may not be associated with p53, p14^ARF^, and p16^INK4A^. However, we did not rule out the involvement of RhoA in our experiment. In addition, the effects of atorvastatin might be similar to those of lovastatin and simvastatin on inducing senescence in different cancer types, and the different mechanisms are probably dependent on the type of statin and/or cancer cells.

Statins inhibit the mevalonate pathway to decrease cholesterol synthesis, and their main effect on tumor cells is mediated by targeting the mevalonate synthesis pathway^35^. Moreover, our previous study demonstrated that simvastatin-induced G0/G1 arrest in HCC cells was p53 independent and mediated through inactivation of the STAT3/Skp2 axis via inhibition of the mevalonate pathway^13^. Similarly, a recent study indicated that inhibiting STAT3 activation and hTERT expression in HCC cells by upregulating the expression of growth arrest and DNA damage 45G induced cellular senescence independent of p53, p14, and p16^36^. We hypothesized that atorvastatin inhibits the STAT3/hTERT axis to induce cellular senescence, which should also be mediated by suppression of the mevalonate pathway in both p53-wild-type HepG2 and p53-null Hep3B cells. Therefore, the antitumor effects of statins, including the induction of cellular senescence, might be independent of the p53 status in cancer cells.

Previous studies have indicated that a high level of IL-6 is associated with HCC and plays an important role during HCC tumor initiation, progression, and recurrence. IL-6 is considered a tumor biomarker, and blocking the IL-6 signaling pathway is considered a therapeutic approach in HCC^25–27^. In addition, based on a population-based prospective cohort study, statin treatment was reported to suppress the production of proinflammatory cytokines, such as IL-6 and IL-8, in hypercholesterolemic patients and to reduce the risk of developing HCC in a dose-dependent manner^37,38^. Statins were also found to reduce IL-6 production in a human vascular SMC/human mononuclear cell coculture model^28^. Consistent with these reports, we observed that atorvastatin not only reduced the expression of IL-6 but also induced senescence in HCC cells (Fig. 3a, b). Although IL-6 is a major SASP cytokine secreted from senescent cells^6,39,40^, recent evidence has indicated that specific types of senescence may display distinct SASP patterns. For example, mitochondrial dysfunction-associated senescence (MiDAS) has been reported to decrease the NAD^+^/NADH ratio; activate AMPK and p53; and subsequently limit the expression of IL-1, IL-6, and IL-8^41^. Interestingly, emerging evidence has indicated that statins impair mitochondrial dysfunction^42^. Atorvastatin has been suggested to damage mitochondrial function in human pancreatic islets and rat β cells^43^. Thus atorvastatin may also impair mitochondrial dysfunction and induce MiDAS to inhibit IL-6 expression in HCC cells. However, whether atorvastatin-induced senescence is mediated by mitochondrial dysfunction and how MiDAS is connected to the IL-6/STAT3/TERT axis in HCC cells requires further study.

Many studies have reported that proinflammatory cytokines are involved in cancer development and promote invasive behavior and stem-like characteristics in cancer cells. A recent study stated that IL-6 stimulation enhanced STAT3 binding to the hTERT promoter region and increased telomerase activity^44,45^. Here we found that atorvastatin not only inhibited STAT3 phosphorylation and TERT protein expression (Fig. 4b) but also decreased telomerase activity in HepG2 cells (Fig. 4c), and these inhibitory effects were reversed by exogenous supplementation of IL-6 (Fig. 5a). We further observed that STAT3C expression maintained higher levels of TERT expression and telomerase activity in atorvastatin-treated HepG2 cells (Fig. 5b–e). Moreover, either supplementation with exogenous IL-6 or overexpression of STAT3C blocked atorvastatin-induced senescence in HepG2 cells. STAT3C-expressing HepG2 cells maintained relatively higher levels of IL-6 after atorvastatin treatment (Fig. 5f). According to these findings, we suggested that atorvastatin decreased IL-6 production to block the IL-6 autocrine loop, leading to diminished binding of activated STAT3 to the hTERT promoter and ultimately decreasing telomerase activity, which causes cellular senescence (Fig. 6f). Thus we concluded that atorvastatin induced cellular senescence by suppressing the IL-6 autocrine loop via inhibition of STAT3 activation, which is crucial for TERT expression in order to maintain HCC cell immortality.

Consistent with the in vitro findings, atorvastatin treatment inhibited tumor growth and reduced tumor volumes in a HepG2 xenograft mouse model. We showed that atorvastatin treatment decreased the levels of IL-6, p-STAT3, and TERT but increased β-gal expression and SA-β-gal activity in tumor lesions. Moreover, poor overall survival of HCC patients was correlated with increased IL-6 and hTERT expression. This in vivo study and clinical evidence confirmed the hypothesis of atorvastatin-induced senescence and the crucial role of the IL-6/STAT3/TERT signaling pathway in HCC progression in vivo. These data provide a novel mechanism explaining the anticancer effect of atorvastatin through the induction of cellular senescence in HCC cells and indicate that atorvastatin-induced senescence may be important in reducing the risk and recurrence of HCC in the clinic.

## Materials and methods

### Reagents and antibodies

TRIzol reagent was obtained from Invitrogen (Carlsbad, CA, USA). Atorvastatin was purchased from Cayman (Ann Arbor, MI, USA). Recombinant human IL-6 and a human IL-6 Standard ABTS enzyme-linked immunosorbent assay (ELISA) Development Kit were purchased from Peprotech (Rocky Hill, NJ, USA). Trypan blue and propidium iodide (PI) were obtained from Sigma (St. Louis, MO, USA). Antibodies against p53, p21, p27, p14^ARF^, p16^INK4a^, p-STAT3, STAT3, β-gal, and β-actin were purchased from Cell Signaling Technology (Danvers, MA, USA). Antibodies against TERT were purchased from EMD Millipore (Temecula, CA, USA).

### Cell culture

The human hepatoma cell lines HepG2 and Hep3B were cultured in Dulbecco**’**s modified Eagle’s medium supplemented with 10% fetal bovine serum (Invitrogen, Carlsbad, CA) in a humidified atmosphere of 5% CO_2_ at 37 °C.

### Cell viability and cell counting

HepG2 and Hep3B cells treated with atorvastatin were assessed for viability measured with a Cell Counting Kit-8 Assay Kit (Sigma). The absorbance at 450 nm was detected with an ELISA plate reader (PerkinElmer, Waltham, MA, USA). To calculate the percentages of dead and live cells, we used the trypan blue exclusion method and a hemocytometer to determine the number of surviving cells. The results are expressed as percentages of the control.

### Cell cycle analysis by flow cytometry

Atorvastatin-treated cells were washed with phosphate-buffered saline (PBS), fixed with 70% ice-cold ethanol at 4 °C overnight, and stained with 20 µg/ml PI at 37 °C for 30 min. The cell cycle distribution was assessed on a flow cytometer (FACS Calibur BD Flow Cytometer, San Jose, CA, USA).

### Reverse transcription–polymerase chain reaction (PCR)

Total RNA was extracted using TRIzol reagent and synthesized into complementary DNA (cDNA) using a Transcriptor First Strand cDNA Synthesis Kit (Clontech, Mountain View, CA, USA). The target genes were amplified by PCR using the following specific primers: TERT, forward 5′-GAACTTGCGGAAGACAGTGG-3′ and reverse 5′-ATGCGTGAAACCTGTACGCCT-3′; IL-6, forward 5′-AGGAGACTTGCCTGGTGAAA-3′ and reverse 5′-AAAGCTGCGCAGAATGAGAT-3′; and GAPDH (endogenous control), forward 5′-ACCACAGTCCATGCCATCAC-3′ and reverse 5′-TCCACCACCCTGTTGCTGTA-3′. PCR was completed through the following steps: step 1, one cycle at 94 °C for 10 min; step 2, 28 cycles at 94 °C for 30 s, 52 °C for 30 s, and 72 °C for 1 min; and step 3, one cycle at 72 °C for 10 min. TEMPase Hot Start DNA Polymerase (Ampliqon, Hamburg) was used for PCR amplification, and the products were separated on 1.5% agarose gels.

### SA-β-gal staining

SA-β-gal staining was performed using a staining kit according to the manufacturer’s instructions (Cell Signaling Technology). β-Gal-positive cells were assessed under a microscope for the development of blue color, and randomly chosen fields were imaged under a light microscope through a ×400 objective. The percentage of senescent cells was calculated as follows: (the number of β-gal-positive cells/the number of total cells in a field) × 100%.

### Telomerase activity

Telomerase activity was measured with a TRAPeze® RT Telomerase Detection Kit according to the manufacturer’s instructions (EMD Millipore, Temecula, CA, USA), and the cell lysate of each sample was subjected to amplification using a real-time PCR system. Quantitative values of telomerase activity were obtained from a standard curve generated from dilutions of the TSR8 control template provided in the kit.

### Plasmid DNA transfection

Cells were transfected with lentiviral vectors expressing constitutively active STAT3-GFP (Addgene, Cambridge, MA, USA) using Lipofectamine 2000 transfection reagent (Invitrogen, Ann Arbor, MI, USA) according to the manufacturer’s instructions. After 24 h, cells were treated with atorvastatin for 72 h and harvested for immunoblot analysis.

### Immunoblot analysis

Cell pellets from each sample were lysed in PRO-PREP protein extraction solution (iNtRON, Taipei, Taiwan) containing protease inhibitor cocktail. Sample proteins were separated on an 8% or 12% sodium dodecyl sulfate–polyacrylamide gel and then transferred to equilibrated polyvinylidene difluoride membranes. After the membranes were blocked, they were incubated with primary antibodies at 4 °C overnight followed by secondary antibodies at room temperature for 2 h. Immunoreactivity was detected with an enhanced chemiluminescence system with β-actin serving as the loading control.

### Tumor growth in a xenograft mouse model

Male BALB/c nude mice (6–8 weeks old) were purchased from the National Laboratory Animal Center (NLAC). HepG2 cells (1.0 × 10^7^) in 0.2 ml of medium were subcutaneously injected into the right flanks of mice. Tumor growth was monitored three times weekly, and treatment was initiated when the mean tumor volume reached approximately 100 mm^3^. Mice were subjected to intraperitoneal injection of vehicle or atorvastatin (20 mg/kg body weight) twice daily. Tumor volumes were measured every other day with calipers and calculated by the following formula: length × (width)^2^ × 0.5. Tumor tissues were collected and weighed individually. All animal care and experimental procedures were approved by the Committee for Animal Experiments, National Chung Hsing University, Taichung, Taiwan (approval document La-1071593).

### Histological analysis and immunohistochemical (IHC) staining

Tumor tissues of mice with HCC were fixed in formalin, embedded in paraffin, and sectioned for IHC. Sections were stained with primary antibodies specific for IL-6, p-STAT3, TERT, and β-gal. Images of the sections were acquired on an inverted microscope.

### β-Galactosidase experiments in tissue sections

The detection of senescent cells was performed using a Senescence Detection Kit, which is designed to histochemically detect SA-β-Gal activity in tissue sections. First, the frozen tissue sections were fixed with 0.5 ml of fixative solution for 10–15 min at room temperature. After PBS washes, the tissue sections were incubated in the staining solution mix (staining solution, staining supplement, and 20 mg/ml X-Gal in dimethyl sulfoxide) at 37 °C overnight in the absence of CO_2_. Photos of the slides were taken with an inverted microscope.

### Statistical analysis

For all assay conditions, three independent experiments with technical duplicates or triplicates were conducted. Data were analyzed with Student’s *t* test, and significant differences were inferred at a *p* value of 0.05. Data are expressed as means ± S.E.M. of three independent experiments, and significant differences are expressed as follows: *, #, ×, and &—*p* < 0.05; **, ##, ××, and &&—*p* < 0.01; and ***, ###, ×××, and &&&—*p* < 0.001.

## Supplementary information

Supplementary Figure legend

Supplementary Figure 1A

Supplementary Figure 1B

Author contribution form
