# Supplementary material for: Atorvastatin-induced senescence of hepatocellular carcinoma is mediated by downregulation of hTERT through the suppression of the IL-6/STAT3 pathway
Source: Cell Death Discov. 2020 Mar 30;6:17. doi: 10.1038/s41420-020-0252-9 (PMC7105491; doi:10.1038/s41420-020-0252-9)
Supplement: Supplementary file 1 — Supplementary Figure legend [file 41420_2020_252_MOESM1_ESM.doc]

**Supplementary Figure 1 Atorvastatin induced G0/G1 phase arrest in HCC cells.** HepG2 **(a)** and Hep3B **(b)** cells were treated with atorvastatin (0, 5, 10, 20 or 40 μg/ml) for 48 or 72 h, and the cell cycle distribution was then analyzed with PI staining and flow cytometry.
