# Supplementary figures and images for: Atorvastatin-induced senescence of hepatocellular carcinoma is mediated by downregulation of hTERT through the suppression of the IL-6/STAT3 pathway
Source: Cell Death Discov. 2020 Mar 30;6:17. doi: 10.1038/s41420-020-0252-9 (PMC7105491; doi:10.1038/s41420-020-0252-9)

## HepG2

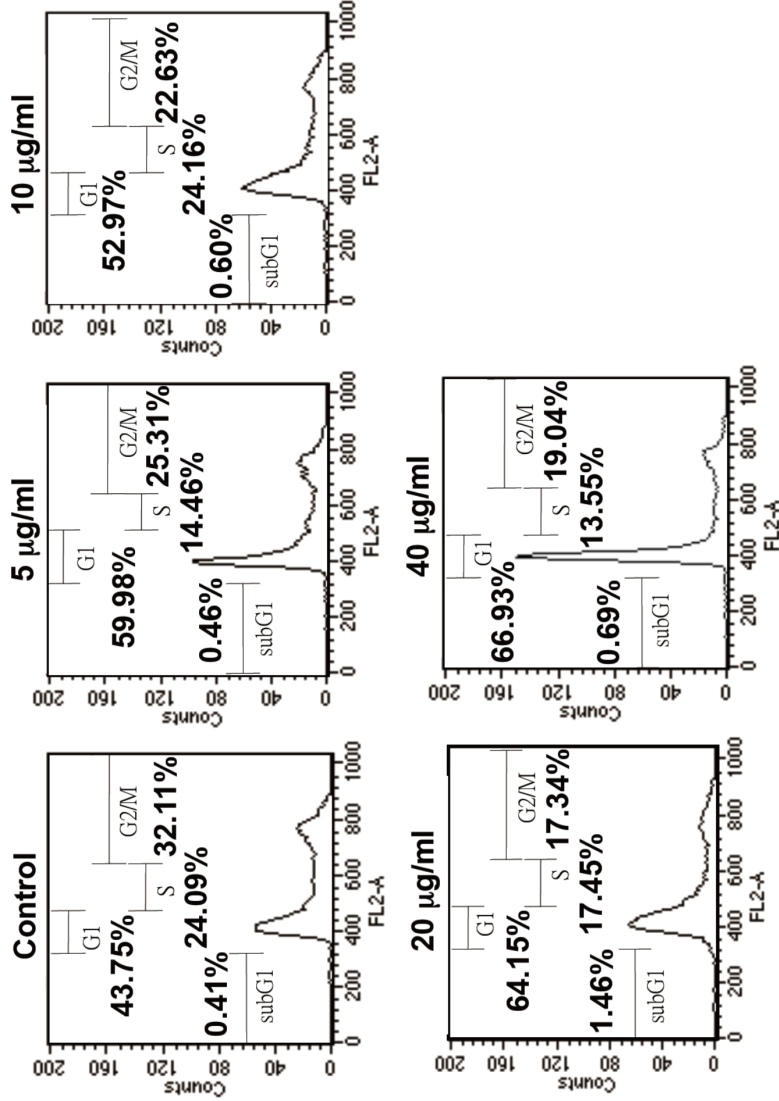

PI Staining (48 h)

## HepG2

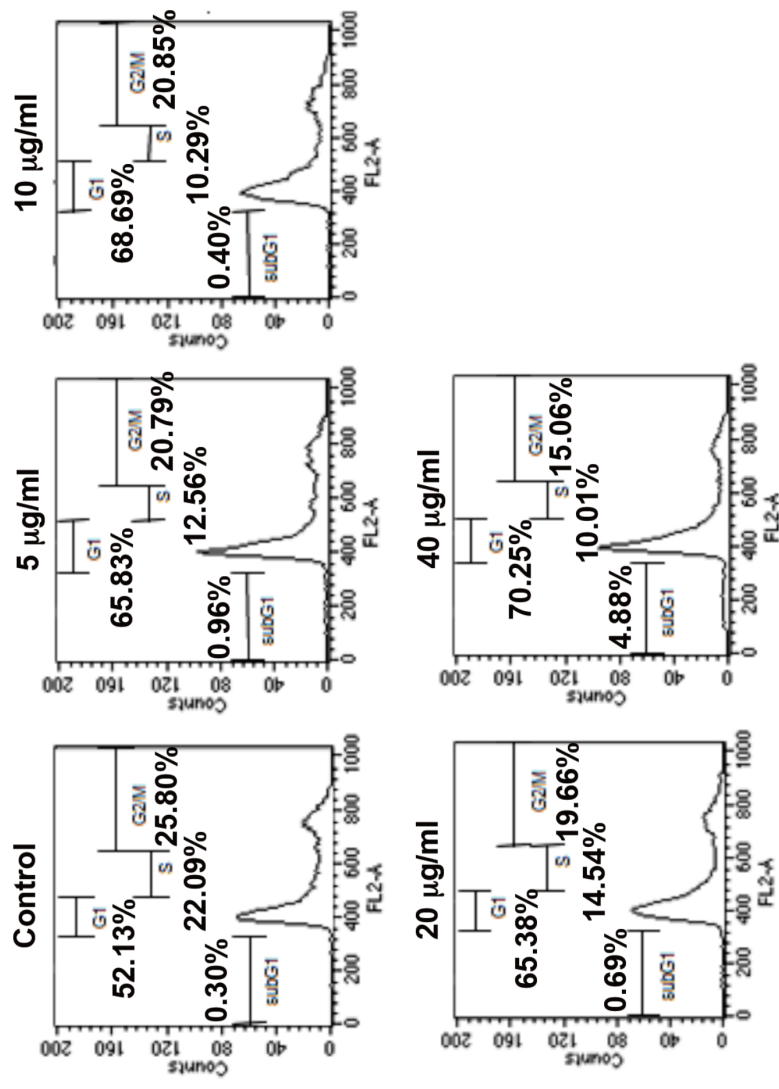

PI Staining (72 h)

Supplement: Supplementary file 2 — Supplementary Figure 1A [file 41420_2020_252_MOESM2_ESM.pdf]

# Hep3B

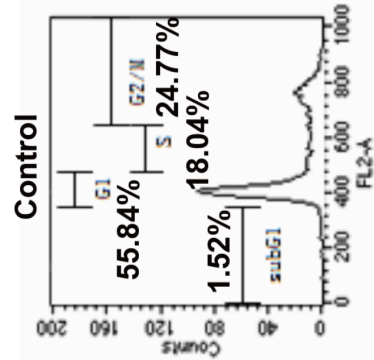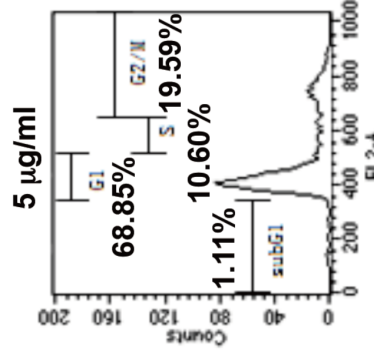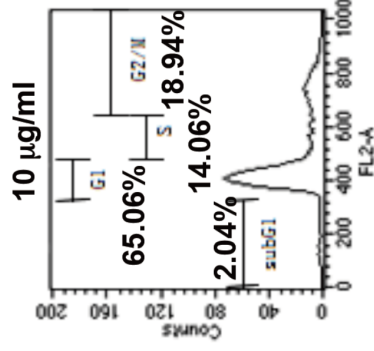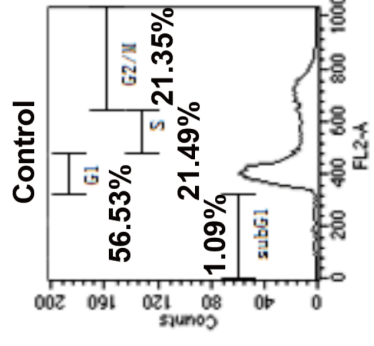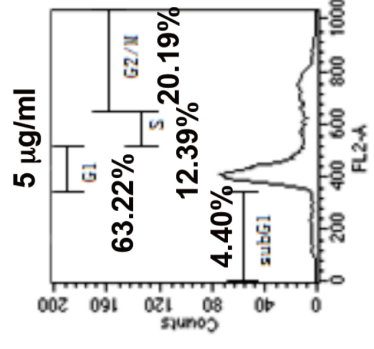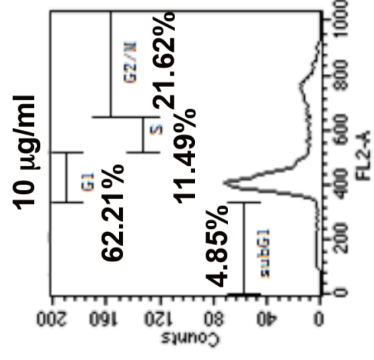

# 20 µg/ml

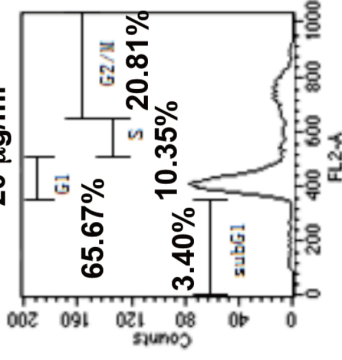

# 40 µg/ml

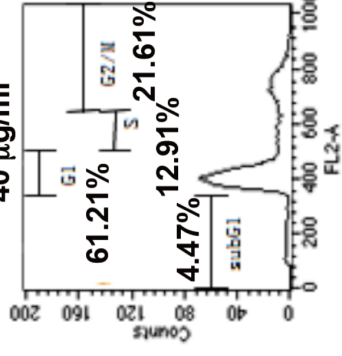

# 20 µg/ml

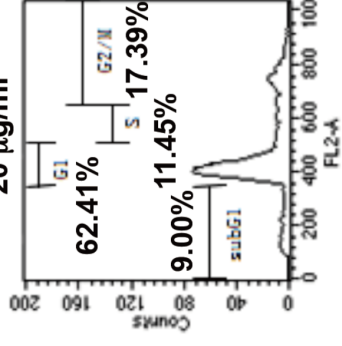

# 40 µg/ml

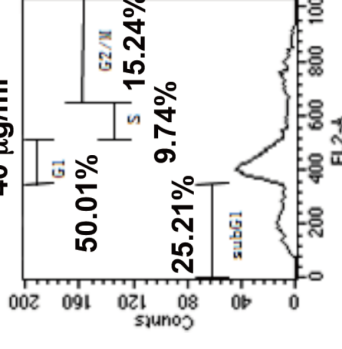

PI Staining (48 h)

PI Staining (72 h)

Supplement: Supplementary file 3 — Supplementary Figure 1B [file 41420_2020_252_MOESM3_ESM.pdf]
