# Supplementary material for: Atorvastatin-induced senescence of hepatocellular carcinoma is mediated by downregulation of hTERT through the suppression of the IL-6/STAT3 pathway
Source: Cell Death Discov. 2020 Mar 30;6:17. doi: 10.1038/s41420-020-0252-9 (PMC7105491; doi:10.1038/s41420-020-0252-9)
Supplement: Supplementary file 4 — Author contribution form [file 41420_2020_252_MOESM4_ESM.pdf]

DECLARATION OF CONTRIBUTIONS TO ARTICLE

ADMC

Manuscript Number: CDDISCOVERY-19-0809-T

Journal Name: *Cell Death Discovery* (the 'Journal')

Proposed Title of the Contribution:

Atorvastatin-induced senescence of hepatocellular carcinoma is mediated by downregulation of hTERT through the suppression of the IL-6/STAT3 pathway (the 'Contribution)

Author(s):

Sin-Ting Wang, Shi-Wei Huang, Kuang-Ting Liu, Teng-Yu Lee, Jeng-Jer Shieh*, Chun-Ying Wu* (the 'Authors')

For all *CDDis*covery articles, each person named as an author in the published version must be able to show he or she has contributed substantially to the article.

Authorship credit should be based on 1) substantial contributions to conception and design, acquisition of data, or analysis and interpretation of data; 2) drafting the article or revising it critically for important intellectual content; and 3) final approval of the version to be published. Authors should meet conditions 1, 2 and 3.

Any person who cannot be shown to have made a substantial contribution to the article cannot be listed as an author in the final version. The name of any person who is deemed to have made a minor contribution can, however, appear in the Acknowledgments section of the article.

Please complete the table below to indicate the contributions of all named authors to the manuscript.

Author Full Name:	Specification of Contribution to the Manuscript:
Sin-Ting Wang	progressed the in vitro and in vivo studies, and wrote this manuscript
Shi-Wei Huang	progressed the in vivo studies and experimental designs
Kuang-Ting Liu	progressed the SA-β-Gal activity in frozen tissue sections and immunohistochemical staining
Teng-Yu Lee	provided advice on clinical opinions; provided research funding
Jeng-Jer Shieh	designed and progressed the whole study, and edited this manuscript
Chun-Ying Wu	provided advice on clinical opinions; provided research funding

Please complete the table below to indicate the contributions of all named authors to the figures.

Figure 1:

Sin-Ting Wang

Figure 2:

Sin-Ting Wang

Figure 3:

Sin-Ting Wang

Figure 4:

Sin-Ting Wang

Figure 5:

Sin-Ting Wang

Figure 6:

Fig.6D, 6E, Kuang-Ting Liu;
Others:Sin-Ting Wang, Shi-Wei Huang

Signed for and on behalf of the Author(s):	Print Name:	Date:
	Jeng-Jer Shieh	2019/11/18
